# Awareness of hepatitis C knowledge among four high-risk populations in Xiamen, China: a cross-sectional study

**DOI:** 10.1186/s12879-026-13862-x

**Published:** 2026-06-20

**Authors:** Jian-hang Xue, Chun-xiang Cai, Ran Ke, Gui-lin Ma, Hui-wen Deng

**Affiliations:** 1 Division of Tuberculosis and HIV/AIDS Prevention and Control, Xiamen Center for Disease Control and Prevention, Xiamen, Fujian China; 2 Division of Disinfection and Vector-borne Infectious Disease Prevention and Control, Xiamen Center for Disease Control and Prevention, Xiamen, Fujian China

**Keywords:** Hepatitis C, Knowledge awareness, High-risk populations, China

## Abstract

**Introduction** Hepatitis C virus (HCV) infection remains a severe global public health challenge. Sufficient HCV knowledge among high-risk populations is essential to achieve the WHO 2030 goal of eliminating viral hepatitis. Comparative evidence on HCV knowledge awareness and its demographic determinants across men who have sex with men (MSM), drug users (DUS), female sex workers (FSW), and invasive procedure patients is scarce in Fujian Province. This study aimed to investigate the status of HCV knowledge awareness and explore its associated factors among the four high-risk populations in Xiamen, China.

**Methods **A hospital- and community-based cross-sectional study was performed among 1621 valid participants. A standardized questionnaire developed by the Chinese Center for Disease Control and Prevention was adopted to collect sociodemographic information and HCV knowledge. Participants scoring ≥ 6 points in the 8 knowledge-related questions were defined as having adequate awareness level. The Chi‑square test and multivariable logistic regression analysis were used to compare intergroup differences and identify factors associated with adequate HCV knowledge.

**Results** The overall HCV knowledge awareness rate was 76.5%, with significant differences across groups: MSM 91.0%, FSW 87.7%, DUS 75.3%, and invasive procedure patients only 51.9% (*χ*^*2*^ = 295.5, *P* < 0.001). Obvious knowledge gaps were identified in HCV curability among MSM and DUS, infection risk of novel psychoactive substance use among DUS, and misconceptions about occult infection and transmission routes among invasive procedure patients. Multivariable logistic regression revealed that college education or above was positively associated with adequate HCV knowledge in MSM (OR = 13.729, 95%*CI*: 4.030-46.767, *P*<0.001), DUS (OR = 16.201, 95%*CI*: 8.874–29.578, *P*<0.001), and invasive procedure patients (OR = 1.741, 95%*CI*: 1.074–2.822, *P* = 0.024). For FSW, aged ≥ 40 years (OR = 2.205, 95%*CI*: 1.091–4.457, *P* = 0.028) and Fujian household registration (OR = 2.696, 95%*CI*: 1.330–5.467, *P* = 0.006) were independent protective factors for better HCV knowledge.

**Conclusions** Substantial disparities in HCV knowledge awareness exist among the four high-risk populations in Xiamen, with invasive procedure patients and DUS being the most disadvantaged groups. Education level, age and household registration were significantly associated with HCV knowledge awareness. Healthcare system-based interventions are crucial for invasive procedure patients, including preoperative HCV counseling, electronic medical record screening prompts, and clinical staff training. Tailored and differentiated health education interventions targeting elderly, migrant and low-education populations are urgently needed to fill knowledge gaps, block HCV transmission, and facilitate the achievement of national and regional hepatitis C elimination targets by 2030.

**Supplementary Information:**

The online version contains supplementary material available at https://doi.org/10.1186/s12879-026-13862-x.

## Introduction

Hepatitis C is a chronic infectious disease induced by the hepatitis C virus (HCV), with transmission predominantly occurring through blood exposure and sexual contact [1]. Without intervention, HCV infection may advance to life-threatening complications, including liver cirrhosis and hepatocellular carcinoma [2]. HCV infection continues to pose a substantial global public health burden. According to the World Health Organization (WHO), approximately 47 million individuals worldwide were living with chronic HCV infection in 2024, corresponding to a global prevalence rate of 0.6%. Around 900,000 incident HCV cases and 240,000 HCV-associated mortalities were documented across the globe in 2024, and the majority of these deaths stemmed from liver cirrhosis and hepatocellular carcinoma. The global epidemiological burden of HCV infection exhibits distinct geographical clustering, and China is listed among the top ten countries bearing the heaviest HCV-related disease burden across the world [3]. Epidemiological data from Fujian Province during 2015–2022 showed a total of 18,712 newly reported HCV cases, with an overall significant downward trend (annual percent change = − 9.19%). However, substantial regional disparities exist in Fujian Province. Notably, Xiamen was one of the few cities in Fujian that showed no statistically significant declining trend. This indicates persistent HCV transmission risks in Xiamen [4].

Against such a context, the Global Health Sector Strategy on Viral Hepatitis (2016–2021), endorsed by the 69th World Health Assembly, established the overarching goal of eliminating viral hepatitis as a prominent public health threat by 2030 [5]. In line with this global initiative, China released the Healthy China Initiative (2019–2030) and the National Action Plan for Eliminating the Public Health Burden of Hepatitis C (2021–2030) in 2019 and 2021, respectively, to facilitate the attainment of the WHO’s 2030 viral hepatitis elimination targets [6–7].

The advent of well-tolerated direct-acting antivirals (DAAs) has markedly optimized therapeutic outcomes for HCV infection and curtailed population-level HCV transmission [8]. Currently, chronic hepatitis C is universally acknowledged as a curable infectious disease. Standardized DAA regimens enable viral elimination within a concise 12-week treatment course, with sustained virological response (SVR) rates exceeding 95% [9]. Although DAAs show prominent therapeutic effects and global HCV elimination targets have been clearly established, widespread insufficient knowledge of HCV prevention, transmission and clinical management exists in general and high-risk populations. This situation greatly impedes timely screening, early diagnosis, and standardized referral and treatment services [10–13]. Limited health literacy concerning HCV results in undiagnosed occult infections and persistent viral transmission, thereby impeding the advancement of national and global hepatitis C elimination strategies.

High-risk groups serve as the core reservoir for HCV transmission, new infections and endemic occult infections, mainly encompassing men who have sex with men (MSM), drug users (DUS), female sex workers (FSW) and invasive procedure patients. These groups bear an excessively heavy burden of HCV exposure and infection [3, 13–14]. Restricted by insufficient health awareness, unfavorable health perceptions, socioeconomic constraints, social stigma and limited access to medical resources, they encounter various obstacles throughout the full-spectrum HCV care pathway from initial screening and diagnosis to SVR attainment [15–18]. However, most current researches only target one single population [19–21]. There is still a lack of comparative analysis on HCV awareness status and relevant influencing factors across the four high-risk population, particularly in Fujian Province of China.

This study aimed to investigate the current status of HCV knowledge awareness among MSM, DUS, FSW, and invasive procedure patients in Xiamen, Fujian Province, and to examine factors associated with HCV knowledge awareness. The findings are expected to provide evidence for the development of targeted health education and optimized HCV prevention and control strategies for high-risk populations, thereby facilitating the achievement of regional and national hepatitis C elimination goals.

## Methods

### Study design

A cross-sectional study was conducted to systematically investigate the HCV knowledge awareness level and associated influencing factors among four high-risk populations in Xiamen, Fujian Province. This research was implemented in strict accordance with the national hepatitis C knowledge survey specification issued by the National Center for AIDS/STD Control and Prevention and the investigation scheme of Fujian Province. Consistent with previous multicenter HCV epidemiological surveys in China, standardized questionnaires, unified recruitment criteria, and rigorous quality control procedures were applied throughout the field investigation. The four targeted populations included MSM, FSW, DUS, and invasive procedure patients. This study aimed to clarify intergroup disparities in HCV cognition and provide evidence for regionally precise hepatitis C elimination strategies.

### Study participants

1. Inclusion Criteria

 Participants were required to meet the following general inclusion criteria: aged 15–65 years; residents or floating population residing in Xiamen for more than six months; capable of independent thinking and verbal communication; and voluntarily participating in the survey with signed informed consent. Based on the national classification criteria for high-risk populations, the specific inclusion definitions for the four subgroups were described as follows: (1)MSM: men who have had insertive oral or anal homosexual intercourse in the past year; (2)FSW: women engaged in commercial sex transactions at present; (3)DUS: individuals who abuse illicit drugs, including heroin, cocaine, opium, cannabis, morphine, methamphetamine, ketamine, ecstasy, and yaba via oral administration, inhalation, or injection; (4)Invasive procedure patients: individuals who have undergone invasive medical procedures such as endoscopy, minimally invasive surgery, dental treatment, and invasive cosmetic procedures in medical institutions. 

2. Exclusion Criteria

Individuals with mental disorders, cognitive impairment, severe physical illness, language communication disorders, and those who were unwilling to cooperate with face-to-face interviews were excluded from this study.

### Sample size and sampling strategy

The sample size for each subgroup was determined based on the requirements of the national and provincial implementation schemes, and was also calculated statistically. The sample size was computed using the formula for cross sectional studies: $n = \frac{{{Z}^2}\times{P(1-P)}}{{d}^2}$ . At a 95% confidence level (Z=1.96), with an anticipated awareness rate (P) of 50% (to achieve a conservative estimate), and a margin of error (d) of 5%, a minimum sample size of 385 was necessary. To account for potential non-responses and ensure robustness, the target was set at 400 participants per group. For MSM, DUS, and FSW, participants were recruited through local community-based organizations (CBOs) and national AIDS sentinel surveillance sites. For invasive procedure patients, consecutive sampling was employed at designated sentinel hospitals until the required sample size was reached.

### Questionnaire

The questionnaire was officially formulated by the Chinese Center for Disease Control and Prevention (China CDC). The full English versions of the questionnaires are provided as a supplementary file. The questionnaire consisted of two sections. The first section collected basic sociodemographic information, including survey region, gender, age, marital status, residential address, ethnicity, and educational level. The second section consisted of eight items concerning hepatitis C prevention and treatment knowledge, covering the occult infection characteristics, transmission routes, daily contact infection risks, disease hazards, and curability of HCV. Each question was designed with three response options: “True”, “False”, and “Do not know”. Corresponding questionnaires were separately applied to different subgroups, including the MSM-specific questionnaire, DUS-specific questionnaire, FSW-specific questionnaire, and general-population questionnaire for invasive procedure patients. Respondents obtained one point for each correct answer, and the total knowledge score ranged from 0 to 8 points. Participants with a total score of 6 points or higher were defined as having adequate HCV-related knowledge. Immediately after the completion of the entire questionnaire, investigators informed each participant of the correct answers to the knowledge items for health education purposes.

### Field investigation

All investigators received standardized pre-investigation training regarding questionnaire specifications, communication skills, and ethical norms. A one-to-one anonymous face-to-face interview was performed to avoid information bias. After each questionnaire was completed, investigators immediately checked for missing items and logical errors. Unqualified questionnaires were eliminated on-site to guarantee data integrity.

### Statistical analysis

The data were entered into a database using EpiData V.3.1 by two researchers independently to ensure accuracy. Statistical analysis was performed through SPSS V20.0. Qualitative data were described using frequency. To compare differences between groups, the Chi-squared test was used. A multivariable logistic regression model was used to identify factors associated with adequate HCV knowledge of participants’ awareness of knowledge of hepatitis C prevention and treatment. Variable selection was performed using a backward stepwise method based on univariate analysis results (variables with *P* ≤ 0.05 in univariate Chi-square test were included in the multivariable model). Categorical variables were treated as dummy variables with reference groups defined as follows: age (< 40 years as reference), education level (below college education as reference), household registration (non‑Fujian province as reference), and marital status (unmarried/single/divorced/widowed as reference). *P* ≤ 0.05 was considered statistically significant. Consistent with standard epidemiological practice, an OR > 1 indicates a positive association with adequate HCV knowledge, while an OR < 1 indicates a negative association with adequate HCV knowledge.

### Ethical considerations

This study was reviewed and approved by the Ethics Committee of the National Center for AIDS/STD Control and Prevention, China CDC (Approval number X210827665). The study was conducted in accordance with the Declaration of Helsinki.

## Result

### Demographic characteristics

In this survey, a total of 1650 participants were enrolled, and 1621 of them finished the investigation questionnaires, with an effective response rate of 98.2%. Demographic characteristics of participants are summarized in Table 1. Participants comprised four high-risk populations: 402 MSM, 401 DUS, 413 FSW, and 405 invasive procedure patients. The mean age was 38 years (95%*CI*: 29–51 years). In total, 984 (60.7%) participants were male, and 637 (39.3%) were female. About marital status, 1,128 (69.6%) were married, 384 (23.7%) were single, and 109 (6.7%) were divorced or widowed. The majority of respondents were of Han ethnicity (96.2%) and held household registration in Fujian Province (74.0%). In terms of educational level, 38.0% had a college education or higher, followed by senior high school or technical secondary school (27.8%), junior high school (21.4%), primary school (10.4%), and illiteracy (2.3%).

**Table 1:** Baseline characteristics of study participants (*n* = 1621)

Characteristics	Number (%)
Population group	
MSM	402 (24.8)
DUS	401 (24.7)
FSW	413 (25.5)
Invasive procedure patients	405 (25.0)
Gender	
Male	984 (60.7)
Female	637 (39.3)
Age group (years)	
<40	868 (53.5)
≥ 40	753 (46.5)
Marital status	
Married	1128 (69.6)
Single	384 (23.7)
Divorced or widowed	109 (6.7)
Ethnic group	
Han	1559 (96.2)
Other groups	62 (3.8)
Residence address	
Fujian Province	1199 (74.0)
Other Provinces	422 (26.0)
Education level	
Illiteracy	38 (2.3)
Primary school	169 (10.4)
Junior high school	347 (21.4)
Senior high school or technical secondary school	451 (27.8)
College education or higher	616 (38.0)

### Knowledge status of HCV among the four high-risk populations

The overall awareness rate of HCV prevention and control knowledge reached 76.50% (1240/1621). Specifically, the awareness rate was 91.0% (366/402) among MSM, 75.3% (302/401) among DUS, 87.7% (362/413) among FSW, and 51.9% (210/405) among invasive procedure patients. The difference in HCV knowledge awareness among the four high-risk populations was statistically significant (*χ²*=295.5, *P* < 0.001), as detailed in Table 2.

MSM had a relatively low awareness rate for the question “Can HCV be curable?”, and a similar pattern was observed among DUS. Additionally, DUS also showed low awareness for “Does the use of novel psychoactive substances increase the risk of HCV infection?” and “Can HCV be transmitted through sexual contact?”. FSW demonstrated satisfactory knowledge levels for the majority of the questions. In contrast, invasive procedure patients had an overall low awareness rate, particularly for four questions: “Can a person infected with HCV be completely asymptomatic and only detectable through testing?”, “Can HCV be curable?”, “Can HCV be transmitted through sexual contact?”, and “Can HCV be transmitted by daily contact such as sharing meals or handshaking?”.

**Table 2:** Knowledge of HCV prevention and control among four high-risk populations [awareness numbers (%)]

Item	MSM (*n* = 402)	DUS (*n* = 401)	FSW (*n* = 413)	Invasive procedure patients (*n* = 405)
Can a person infected with HCV be completely asymptomatic and only detectable through testing? (Yes)	378 (94.0)	396 (98.8)	316 (76.5)	198 (48.9)
Does having multiple sexual partners increase the risk of HCV infection? (Yes)	378 (94.0)	-	348 (84.3)	-
Does consistent and correct condom use reduce the risk of HCV infection and transmission? (Yes)	386 (96.0)	-	373 (90.3)	-
Can HCV be transmitted by sharing injection equipment? (Yes)	381 (94.8)	393 (98.0)	376 (91.0)	308 (76.0)
Can HCV be transmitted by transfusion of contaminated blood or blood products? (Yes)	387 (96.3)	399 (99.5)	384 (93.0)	327 (80.7)
Can HCV be transmitted by tattoos, permanent makeup, or body piercings in unlicensed settings? (Yes)	334 (83.1)	328 (81.8)	390 (94.4)	267 (65.9)
Can chronic HCV infection progress to cirrhosis and/or hepatocellular carcinoma? (Yes)	339 (84.3)	382 (95.3)	379 (91.8)	281 (69.4)
Can HCV be curable? (Yes)	257 (63.9)	202 (50.4)	342 (82.8)	205 (50.6)
Does the use of novel psychoactive substances increase the risk of HCV infection? (Yes)	-	188 (46.9)	-	-
Can HCV be transmitted through sexual contact? (Yes)	-	249 (62.1)	-	211 (52.1)
Can HCV be transmitted by daily contact such as sharing meals or handshaking? (No)	-	-	-	217 (53.6)
Total	366 (91.0)	302 (75.3)	362 (87.7)	210 (51.9)

### Factors associated with awareness of hepatitis C knowledge among four high-risk populations

Table 3 presents HCV awareness rates stratified by demographic characteristics across different high-risk populations. Significant differences in overall HCV knowledge awareness were observed with respect to age, marital status, educational level, and household registration location among the studied populations. Specifically, significant associations were identified between age and educational attainment and HCV awareness in MSM; between age, marital status, educational attainment, and HCV awareness in DUS; between age, marital status, residence address, and HCV awareness in FSW; and between age, educational attainment, and HCV awareness in invasive procedure patients.

**Table 3:** Association of awareness with characteristics among the four high-risk populations

Population group	Characteristics		Total *N* (%)	Awareness *N* (%)	Unawareness *N* (%)	OR (95% CI)	*P*
MSM	Age group (years)	<40	241 (60.0)	226 (93.8)	15 (6.2)	1.0	
		≥ 40	161 (40.0)	140 (87.0)	21 (13.0)	**0.442 (0.221–0.887)**	**0.016**
	Marital status	Others	292 (72.6)	269 (92.1)	23 (7.9)	1.0	
		Married	110 (27.4)	97 (88.2)	13 (11.8)	0.638 (0.311–1.309)	0.150
	Residence address	Others	60 (14.9)	51 (85.0)	9 (15.0)	1.0	
		Fujian Province	342 (85.1)	315 (92.1)	27 (7.9)	2.059 (0.916–4.630)	0.069
	Ethnic Group	Others	10 (0.2)	8 (80.0)	2 (20.0)	1.0	
		Han	392 (99.8)	358 (91.3)	34 (8.7)	2.632 (0.537–12.894)	0.223
	Education level	Others	191 (47.5)	158 (82.7)	33 (17.3)	1.0	
		College education or higher	211 (52.5)	208 (98.6)	3 (1.4)	**14.481 (4.362–48.072)**	**<0.001**
DUS	Age group (years)	<40	308 (76.8)	239 (77.6)	69 (22.4)	1.0	
		≥ 40	93 (23.2)	63 (67.7)	30 (32.3)	0.606 (0.364–1.010)	0.053
	Marital status	Others	319 (80.0)	249 (78.1)	70 (21.9)	1.0	
		Married	82 (20.0)	53 (64.6)	29 (35.4)	**0.514 (0.304–0.868)**	**0.012**
	Residence address	Others	80 (20.0)	60 (75.0)	20 (25.0)	1.0	
		Fujian Province	321(80.0)	242(75.4)	79 (24.6)	1.021(0.580–1.799)	0.942
	Ethnic Group	Others	21 (5.2)	17 (81.0)	4 (19.0)	1.0	
		Han	380 (94.8)	285 (75.0)	95 (25.0)	0.706(0.232–2.150)	0.538
	Education level	Others	152 (37.9)	70 (46.1)	82 (53.9)	1.0	
		College education or higher	249 (62.1)	232 (93.2)	17 (6.8)	**15.987 (8.891–28.745)**	**<0.001**
FSW	Age group (years)	<40	194 (47.0)	159 (82.0)	35 (18.0)	1.0	
		≥ 40	219 (53.0)	203 (92.7)	16 (7.3)	**2.741 (1.420–5.288)**	**0.002**
	Marital status	Others	152 (36.8)	125 (82.2)	27 (17.8)	1.0	
		Married	261 (63.2)	237 (90.8)	24 (9.2)	**2.133 (1.181–3.852)**	**0.011**
	Residence address	Others	240 (58.1)	200 (83.3)	40 (16.7)	1.0	
		Fujian Province	173 (41.9)	162 (93.6)	11 (6.4)	**2.945 (1.465–5.924)**	**0.002**
	Ethnic Group	Others	27 (6.5)	22 (81.5)	5 (18.5)	1.0	
		Han	386 (93.5)	340 (88.1)	46 (11.9)	1.136 (0.406–3.179)	0.807
	Education level	Others	359 (86.9)	313 (87.2)	46 (12.8)	1.0	
		College education or higher	54 (13.1)	49 (90.7)	5 (9.3)	1.440 (0.546–3.082)	0.314
Invasive procedure patients	Age group (years)	<40	125 (30.9)	76 (60.8)	49 (39.2)	1.0	
		≥ 40	280 (69.1)	134 (47.9)	146 (52.1)	**0.592 (0.385–0.909)**	**0.016**
	Marital status	Others	41 (10.1)	26 (63.4)	15 (36.6)	1.0	
		Married	364 (89.9)	184 (50.5)	180 (49.5)	0.590 (0.302–1.150)	0.118
	Residence address	Others	42 (10.4)	21 (50.0)	21 (50.0)	1.0	
		Fujian Province	363 (89.6)	189 (52.1)	174 (47.9)	1.086 (0.573–2.058)	0.800
	Ethnic Group	Others	4 (1.0)	1 (25.0)	3 (75.0)	1.0	
		Han	401 (99.0)	209 (52.1)	192 (47.9)	3.266 (0.337–31.661)	0.284
	Education level	Others	302 (74.6)	144 (47.7)	158 (52.3)	1.0	
		College education or higher	103 (25.4)	66 (64.1)	37 (35.9)	**2.130 (1.329–3.412)**	**0.001**

Multivariable logistic regression analyses were performed to further examine these associations in Table 4. Educational level was consistently associated with higher odds of adequate HCV knowledge across multiple populations. Compared to participants with below-college education, those with a college degree or higher were significantly more likely to be aware of HCV knowledge in the MSM (OR = 13.729, 95%*CI*: 4.030–46.767, *P* < 0.001), DUS (OR = 16.201, 95%*CI*: 8.874–29.578, *P* < 0.001), and invasive procedure patients (OR = 1.741, 95%*CI*: 1.074–2.822, *P* = 0.024). Age and household registration were significantly associated with HCV knowledge awareness among FSW. Participants aged ≥ 40 years had a higher probability of HCV knowledge awareness than those aged<40 years (OR = 2.205, 95%*CI* = 1.091–4.457, *P* = 0.028). Moreover, participants with Fujian household registration showed a higher likelihood of HCV knowledge awareness compared with those from other provinces (OR = 2.696, 95%*CI* = 1.330–5.467, *P* = 0.006).

**Table 4:** Multivariable logistic regression model of awareness of hepatitis C knowledge among the four high-risk populations

Population group	Characteristics		B	SE	OR (95%CI)	*P*
MSM	Age group (years)	<40			1.0	
		≥ 40	−0.156	0.376	0.856 (0.410–1.787)	0.679
	Education level	Others			1.0	
		College education or higher	2.620	0.625	**13.729 (4.030−46.767)**	**< 0.001**
DUS	Marital status	Others			1.0	
		Married	0.062	0.315	1.064 (0.574–1.973)	0.843
	Education level	Others			1.0	
		College education or higher	2.785	0.307	**16.201 (8.874–29.578)**	**< 0.001**
FSW	Age group (years)	<40			1.0	
		≥ 40	0.791	0.359	**2.205 (1.091–4.457)**	**0.028**
	Marital status	Others			1.0	
		Married	0.369	0.341	1.446 (0.741–2.822)	0.280
	Residence address	Others			1.0	
		Fujian Province	0.992	0.361	**2.696 (1.330–5.467)**	**0.006**
Invasive procedure patients	Age group (years)	<40			1.0	
		≥ 40	−0.371	0.230	0.690 (0.440–1.083)	0.107
	Education level	Others			1.0	
		College education or higher	0.555	0.246	**1.741 (1.074–2.822)**	**0.024**

## Discussion

This study enrolled MSM, DUS, FSW, and invasive procedure patients in Xiamen, Fujian Province, to systematically analyze the current status and demographic influencing factors of HCV knowledge awareness. Few domestic studies have conducted comparative analyses across the four aforementioned high-risk populations. Clarifying the level and determinants of HCV knowledge among high-risk groups is critical for designing targeted health education initiatives, interrupting human-to-human viral transmission, and accelerating the attainment of the 2030 global viral hepatitis elimination targets.

The overall HCV knowledge awareness rate across the four high-risk populations was 76.50%, with significant intergroup differences. MSM showed the highest awareness rate of 91.0%, followed by FSW at 87.7% and DUS at 75.3%. Notably, invasive procedure patients had an extremely low awareness rate of only 51.9%, indicating a severe shortage of general cognition regarding HCV. The HCV knowledge levels of MSM and FSW in this study were slightly higher than those reported in previous relevant studies [22–23]. In China, MSM and FSW have long been the core target groups of the integrated HIV and sexually transmitted disease prevention and control system. Routine surveillance data have confirmed that these populations are covered by high-intensity regular health education. Moreover, Viral hepatitis prevention and control services have been strategically integrated into the well-established HIV prevention and intervention system, allowing MSM and FSW to access sustained, standardized HCV-related health education resources [24]. The suboptimal HCV knowledge level among DUS identified in our study is consistent with global evidence regarding drug-using populations. A cross-sectional study among newly onset injection drug users in Germany indicated that 41% of HCV-infected individuals were unaware of their infection status. Likewise, existing research from Australia rated the overall HCV knowledge of people who use drugs as “poor” [25–26]. For DUS, regular venue-based and personalized targeted health education on HCV prevention is strongly recommended. Supported by drug control agencies, compulsory drug rehabilitation facilities and community anti-drug service networks, we should establish a sustainable collaborative mechanism covering health education, voluntary screening as well as standardized referral and treatment. Such measures can effectively elevate HCV-related health literacy and raise their initiative to participate in relevant medical screening.

Invasive procedure patients exhibited the lowest HCV knowledge awareness rate (51.9%) among the four high-risk populations in this study. This result is comparable to previous cross-sectional surveys focusing on surgical and hospitalized patients. For example, a large-scale study conducted in Iraq, reported that merely 19% of surgical patients achieved a good level of HCV knowledge, and up to 63.5% had insufficient cognition of HCV prevention and transmission [27]. As a marginalized high-risk subgroup, invasive procedure patients lack targeted HCV health education, risk notification, and professional screening guidance. Their potential risk of HCV infection is often underestimated by the general public and medical institutions, forming a hidden high-risk gap within the existing hepatitis C prevention and control system. Corresponding optimization of prevention strategies should be implemented urgently. This population should be incorporated into regular HCV health education and screening initiatives, with HCV popular science integrated into community health promotion, admission health education, preoperative health counseling, and postoperative follow-up management. Furthermore, professional training on HCV epidemiology and transmission characteristics should be strengthened among clinical and primary care practitioners. It is essential to establish a full-chain service model covering HCV screening, diagnosis, referral, and standardized treatment for invasive procedure patients, so as to fill the current prevention loopholes.

In terms of knowledge structure, MSM and FSW showed relatively high awareness of HCV transmission routes including multiple sexual partners, unsafe sexual behaviors and blood-borne exposure. This phenomenon can be mainly attributed to the long-term implementation of behavioral interventions and persistent peer health education [28–29]. Nevertheless, our data revealed a prominent knowledge gap among MSM and FSW, namely insufficient awareness that HCV is curable. A Saudi Arabian study reported that merely 47.3% of participants recognized that hepatitis C was curable [30]. Insufficient public understanding of HCV curability may induce unnecessary public fear, exacerbate social prejudice and discrimination against infected individuals, and lead to delayed medical presentation among undiagnosed or confirmed cases, consequently elevating the risk of progressive liver injury. Accordingly, improving public awareness of the curative potential of hepatitis C is essential to facilitate early intervention, interrupt transmission, and reduce the societal healthcare burden [10]. Our results revealed that invasive procedure patients had substantial knowledge deficits regarding the occult nature of HCV infection, transmission routes, infection risks of daily social contact, and available treatment options. Such inadequate knowledge may impede timely referral to systematic follow-up, screening, and treatment, even among individuals incidentally detected via preoperative screening or those asymptomatically infected after invasive procedures. This view is strongly backed by existing evidence. Relevant studies confirm that most people with a history of surgery or invasive interventions lack awareness of long-term HCV follow-up demands, receive inadequate in-hospital health education, and fail to fully realize the importance of regular postoperative re-examination and long-term health management [31–32].

Notably, DUS displayed alarmingly low awareness of HCV infection risks linked to new psychoactive substance (NPS) use. In recent years, the number of traditional injection drug users in China has gradually declined, accompanied by a marked shift in the national drug abuse structure. The prevalence of NPS misuse has continuously increased, reflecting an obvious transition from traditional to emerging drug types [33]. Although NPS are mostly administered via non-injectable routes, the HCV infection burden among this population remains non-negligible. Relevant studies have reported that the HCV prevalence among NPS users ranges from 17.3% to 51.0% [34–35]. Low risk perception weakens self-protection awareness and reduces voluntary screening willingness, hindering early identification of occult HCV infections. This not only accelerates endogenous transmission within drug-using groups but also facilitates spillover to the general population through sexual contact, forming a concealed epidemic chain. Therefore, targeted health education on HCV prevention and risk mitigation should be routinely implemented in entertainment venues and other high-risk settings for new psychoactive substance abuse.

Multivariable regression analysis confirmed that educational level was the factor most consistently associated with HCV knowledge awareness across three high-risk populations. Participants with college education or higher exhibited significantly higher odds of having sufficient HCV-related knowledge, with OR values of 13.729 for MSM, 16.201 for DUS, and 1.741 for invasive procedure patients. This finding is consistent with global evidence demonstrating that higher educational attainment is closely linked to better health literacy. Individuals with higher education exhibit stronger capacities in information recognition, knowledge acquisition, reading comprehension, and active acceptance of infectious disease prevention education, enabling them to develop accurate HCV perceptions and appropriate self-protection behaviors [36–37]. For high-risk subgroups with low educational attainment, traditional text-based health education should be replaced with accessible, easy-to-understand formats such as short videos. Such approaches can lower barriers to knowledge understanding, with priority given to core information regarding HCV transmission routes, characteristics of occult infection, and curability. Among FSW, individuals aged ≥ 40 years (OR = 2.205) and local Fujian residents (OR = 2.696) had higher odds of possessing sufficient HCV-related knowledge. This subgroup has long been continuously exposed to comprehensive STD and HIV health education programs implemented in Fujian Province. Compared with younger migrant FSWs, residents enjoy better accessibility to medical services and higher frequency of exposure to health literacy resources, resulting in longer cumulative intervention duration and more solid knowledge reserves. Future strategies should maintain the existing advantages of routine health education for local FSW, while implementing differentiated and hierarchical interventions that prioritize younger and migrant subgroups to bridge their knowledge gaps. In contrast, the elderly invasive procedure patients group showed markedly poor HCV knowledge levels. This finding highlights an unbalanced coverage of current hepatitis C health education and insufficient outreach targeting older adults. It suggests that future health promotion initiatives should prioritize elderly populations as a key focus of targeted intervention.

Most prior domestic and overseas studies have separately explored HCV-related knowledge within a single high-risk group, including MSM, DUS and FSW. Studies simultaneously including invasive procedure patients for cross-group comparison remain scarce. In particular, large-sample comparative data from Fujian Province are still insufficient. This study filled the regional research gap by recruiting all four core high-risk groups and comparing their HCV knowledge status. Nevertheless, several limitations of this study should be noted. First, the cross-sectional design cannot establish causal relationships between demographic characteristics and HCV knowledge levels. The cross-sectional design limits causal inference between demographic characteristics and HCV knowledge. Observed associations do not imply causation. Secondly, all participants were exclusively enrolled in Xiamen, a single city in Fujian Province. The socioeconomic, healthcare access, and public health intervention landscape in Xiamen may differ from other regions in Southeast China or across the nation. Consequently, the findings may not be fully generalizable to other geographic settings, which limits the external validity of our study. Future multi-center and large-scale comparative investigations covering urban and rural areas, as well as different regions in southeastern China, are required to further verify the present findings and improve the representativeness of conclusions. Third, the four high-risk groups were defined based on the study protocol and reflect different exposure routes rather than equivalent comparison groups. A general community population was not included as a control, since this study focused on intra–high-risk group comparisons to support tailored interventions rather than evaluating differences relative to the general population.

## Conclusions

This study indicated that despite the improvement in HCV-related knowledge awareness among some high-risk groups in Xiamen, marked inter-group differences still exist. Educational attainment, age and household registration were significantly associated with HCV knowledge levels. Insufficient HCV knowledge among invasive procedure patients, coupled with enduring knowledge deficiencies among DUS, has become a key obstacle to reaching regional HCV elimination targets. Targeted and differentiated health education strategies should be formulated according to the characteristics of each high-risk group. For invasive procedure patients healthcare system-based interventions are crucial. These should include integrating standardized HCV knowledge briefings into preoperative counseling and informed consent procedures, utilizing electronic medical record systems to prompt screening and education for patients scheduled for invasive procedures, and training frontline clinical staff to deliver key prevention messages during patient encounters. It is necessary to strengthen grassroots health promotion and popularization of HCV prevention and control, with particular focus on the elderly, migrant populations, and individuals with low educational attainment. Meanwhile, medical institutions should scale up professional health education on hepatitis C to improve the overall health literacy and medical-seeking awareness of high-risk populations. Such initiatives can help curb occult HCV infections and sustained transmission, and ultimately facilitate the achievement of national and regional targets to eliminate viral hepatitis as a major public health threat by 2030.

Tables 1 and 2 are placed at the end of the manuscript owing to their large layout size, and should be inserted at the marked positions in the main text.

## Supplementary Information

Below is the link to the electronic supplementary material.

Supplementary Material 1

## Data Availability

The dataset is not publicly available to protect participant privacy. Data are available from the corresponding author upon reasonable request.

## Abbreviations

HCV: Hepatitis C Virus
MSM: Men Who Have Sex With Men
DUS: Drug Users
FSW: Female Sex Workers
WHO: World Health Organization
DAA: Direct-Acting Antivirals
SVR: Sustained Virological Response
AIDS: Acquired Immunodeficiency Syndrome
STD: Sexually Transmitted Diseases
CBO: Community-Based Organization
NPS: New Psychoactive Substance
