# Supplementary material for: Awareness of hepatitis C knowledge among four high-risk populations in Xiamen, China: a cross-sectional study
Source: BMC Infect Dis. 2026 Jun 20;26:1600. doi: 10.1186/s12879-026-13862-x (PMC13540829; doi:10.1186/s12879-026-13862-x)
Supplement: Supplementary file 1 [file 12879_2026_13862_MOESM1_ESM.pdf]

Supplementary File

Awareness of Hepatitis C Prevention and Control Knowledge Among MSM

A01 Administrative Division Code of Sentinel Site Location □□□□□□

A02 Questionnaire ID (001-999) □□□

1. Demographic Information

B01 Date of Birth Year: ______ Month: ______

B02 Marital Status ① Married ② Single ③ Divorced or widowed

B03 Residence Address ① Native province ② Other provinces (Please specify: ______ Province) ③ Foreign national (Please specify: ______ Country)

B04 Ethnic Group ① Han ② Other (Please specify): ____________

B05 Education Level ① Illiteracy ② Primary school ③ Junior high school ④ Senior high school or technical secondary school ⑤ College education or higher

2. Questions on Hepatitis C Prevention and Control Knowledge

C01 Can a person infected with HCV be completely asymptomatic and only detectable through testing?	① True ② False ③ Do not know
C02 Does having multiple sexual partners increase the risk of HCV infection?	① True ② False ③ Do not know
C03 Does consistent and correct condom use reduce the risk of HCV infection and transmission?	① True ② False ③ Do not know
C04 Can HCV be transmitted by sharing injection equipment?	① True ② False ③ Do not know

C05 Can HCV be transmitted by transfusion of contaminated blood or blood products?	① True ② False ③ Do not know
C06 Can HCV be transmitted by tattoos, permanent makeup, or body piercings in unlicensed settings?	① True ② False ③ Do not know
C07 Can chronic HCV infection progress to cirrhosis and/or hepatocellular carcinoma?	① True ② False ③ Do not know
C08 Can HCV be curable?	① True ② False ③ Do not know

Interviewer Signature: ________ **Supervisor Signature: ________**

Awareness of Hepatitis C Prevention and Control Knowledge Among DUS

A01 Administrative Division Code of Sentinel Site Location □□□□□□

A02 Questionnaire ID (001-999) □□□

1. Demographic Information

B01 Gender ① Male ② Female

B02 Date of Birth Year: ______ Month: ______

B03 Marital Status ① Married ② Single ③ Divorced or widowed

B04 Residence Address ① Native province ② Other provinces (Please specify: ______ Province) ③ Foreign national (Please specify: ______ Country)

B05 Ethnic Group ① Han ② Other (Please specify): ____________

B06 Education Level ① Illiteracy ② Primary school ③ Junior high school ④

Senior high school or technical secondary school ⑤ College education or higher

2. Questions on Hepatitis C Prevention and Control Knowledge

C01 Can a person infected with HCV be completely asymptomatic and only detectable through testing?	① True ② False ③ Do not know
C02 Can HCV be transmitted by sharing injection equipment?	① True ② False ③ Do not know
C03 Can HCV be transmitted by transfusion of contaminated blood or blood products?	① True ② False ③ Do not know
C04 Can HCV be transmitted by tattoos, permanent makeup, or body piercings in unlicensed settings?	① True ② False ③ Do not know
C05 Can chronic HCV infection progress to cirrhosis and/or hepatocellular carcinoma?	① True ② False ③ Do not know
C06 Can HCV be curable?	① True ② False ③ Do not know
C07 Does the use of novel psychoactive substances increase the risk of HCV infection?	① True ② False ③ Do not know
C08 Can HCV be transmitted through sexual contact?	① True ② False ③ Do not know

Interviewer Signature: ________ **Supervisor Signature: ________**

Awareness of Hepatitis C Prevention and Control Knowledge Among FSW

A01 Administrative Division Code of Sentinel Site Location □□□□□□

A02 Questionnaire ID (001-999) □□□

1. Demographic Information

B01 Date of Birth Year: ______ Month: ______

B02 Marital Status ① Married ② Single ③ Divorced or widowed

B03 Residence Address ① Native province ② Other provinces (Please specify: ______ Province) ③ Foreign national (Please specify: ______ Country)

B04 Ethnic Group ① Han ② Other (Please specify): ____________

B05 Education Level ① Illiteracy ② Primary school ③ Junior high school ④ Senior high school or technical secondary school ⑤ College education or higher

2. Questions on Hepatitis C Prevention and Control Knowledge

C01 Can a person infected with HCV be completely asymptomatic and only detectable through testing?	① True ② False ③ Do not know
C02 Does having multiple sexual partners increase the risk of HCV infection?	① True ② False ③ Do not know
C03 Does consistent and correct condom use reduce the risk of HCV infection and transmission?	① True ② False ③ Do not know
C04 Can HCV be transmitted by sharing injection equipment?	① True ② False ③ Do not know
C05 Can HCV be transmitted by transfusion of contaminated blood or blood products?	① True ② False ③ Do not know

C06 Can HCV be transmitted by tattoos, permanent makeup, or body piercings in unlicensed settings?	① True ② False ③ Do not know
C07 Can chronic HCV infection progress to cirrhosis and/or hepatocellular carcinoma?	① True ② False ③ Do not know
C08 Can HCV be curable?	① True ② False ③ Do not know

Interviewer Signature: ________ **Supervisor Signature: ________**

Awareness of Hepatitis C Prevention and Control Knowledge Among Invasive procedure patients

A01 Administrative Division Code of Sentinel Site Location □□□□□□

A02 Questionnaire ID (001-999) □□□

1. Demographic Information

B01 Gender ① Male ② Female

B02 Date of Birth Year: ______ Month: ______

B03 Marital Status ① Married ② Single ③ Divorced or widowed

B04 Residence Address ① Native province ② Other provinces (Please specify: ______ Province) ③ Foreign national (Please specify: ______ Country)

B05 Ethnic Group ① Han ② Other (Please specify): ____________

B06 Education Level ① Illiteracy ② Primary school ③ Junior high school ④ Senior high school or technical secondary school ⑤ College education or higher

2. Questions on Hepatitis C Prevention and Control Knowledge

C01 Can a person infected with HCV be completely asymptomatic and only detectable through testing?	① True ② False ③ Do not know
C02 Can HCV be transmitted by sharing injection equipment?	① True ② False ③ Do not know
C03 Can HCV be transmitted by transfusion of contaminated blood or blood products?	① True ② False ③ Do not know
C04 Can HCV be transmitted by tattoos, permanent makeup, or body piercings in unlicensed settings?	① True ② False ③ Do not know
C05 Can chronic HCV infection progress to cirrhosis and/or hepatocellular carcinoma?	① True ② False ③ Do not know
C06 Can HCV be curable?	① True ② False ③ Do not know
C07 Can HCV be transmitted through sexual contact?	① True ② False ③ Do not know
C08 Can HCV be transmitted by daily contact such as sharing meals or handshaking?	① True ② False ③ Do not know

Interviewer Signature: ________ **Supervisor Signature: ________**
